# Comparison of a coaxial versus non-coaxial liver biopsy technique in an oncological setting: diagnostic yield, complications and seeding risk

**DOI:** 10.1007/s00330-020-07038-7

**Published:** 2020-07-14

**Authors:** Nicos Fotiadis, Katja N. De Paepe, Lawrence Bonne, Nasir Khan, Angela Riddell, Nicholas Turner, Naureen Starling, Marco Gerlinger, Sheela Rao, Ian Chau, David Cunningham, Dow-Mu Koh

**Affiliations:** 1 Department of Radiology, The Royal Marsden NHS Foundation Trust, Downs Rd, Sutton, London, SW2 5PT UK; 2 Department of Medical, The Royal Marsden NHS Foundation Trust, London, UK; 3 Translational Oncogenomics, Institute of Cancer Research, London, UK

**Keywords:** Image-guided biopsy, Liver, Neoplasm seeding, Cancer

## Abstract

**Objectives:**

Percutaneous liver biopsy (PLB) poses specific challenges in oncological patients such as bleeding and tumour seeding. This study’s aim was to compare a coaxial (C-PLB) and non-coaxial (NC-PLB) biopsy technique in terms of diagnostic yield, safety and seeding risk of image-guided PLB techniques in an oncological setting.

**Methods:**

Local research committee approval was obtained for this single-site retrospective study. Patients who underwent a PLB between November 2011 and December 2017 were consecutively included. Medical records were reviewed to determine diagnostic yield and complications. Follow-up imaging was re-reviewed for seeding, defined as visible tumour deposits along the PLB track. Mann-Whitney *U* and chi-squared tests were performed to investigate differences between biopsy techniques in sample number, complications and seeding rate.

**Results:**

In total, 741 patients (62 ± 13 years, 378 women) underwent 932 PLB (C-PLB 72.9% (679/932); NC-PLB 27.1% (253/932)). More tissue cores (*p* < 0.001) were obtained with C-PLB (median 4 cores; range 1–12) compared with NC-PLB (2 cores; range 1–4) and diagnostic yield was similar for both techniques (C-PLB 92.6% (629/679); NC-PLB 92.5% (234/253); *p* = 0.940). Complication rate (9.3%; 87/932) using C-PLB (8.2% (56/679)) was lower compared with NC-PLB (12.3% (31/253); *p* = 0.024). Major complications were uncommon (C-PLB 2.7% (18/679); NC-PLB 2.8% (7/253)); bleeding developed in 1.2% (11/932; C-PLB 1.2% (8/679); NC-PLB 1.2% (3/253)). Seeding was a rare event, occurring significantly less in C-PLB cases (C-PLB 1.3% (7/544); NC-PLB 3.1% (6/197); *p* = 0.021).

**Conclusions:**

C-PLB allows for high diagnostic tissue yield with a lower complication and seeding rate than a NC-PLB and should be the preferred method in an oncological setting.

**Key Points:**

*• A coaxial percutaneous liver biopsy achieves a significant higher number of cores and fewer complications than a non-coaxial biopsy technique.*

*• The risk of tumour seeding is very low and is significantly lower using the coaxial biopsy technique.*

*• In this study*, *a larger number of cores* (*median = 4*) *could be safely acquired using the coaxial technique*, *providing sufficient material for advanced molecular analysis.*

## Introduction

Image-guided percutaneous liver biopsy (PLB) is a commonly used minimally invasive technique to retrieve tissue, usually for diagnostic purposes. In the realm of precision medicine [1, 2], the demand for PLB is increasing as clinical cancer trials mandate advanced genetic and molecular tissue analyses to appropriately select and monitor patients for tailored cancer treatment [3]. Therefore, acquisition of sufficient tissue is essential and usually obtained via PLB at different time points during treatment. Yet, special consideration is needed for the fact that more and sometimes larger tissue cores are required [4], which may increase the complication rate and the discomfort for the patient when using the standard non-coaxial PLB technique (NC-PLB) due to multiple needle passes through the liver capsule. Although several studies have shown that PLB is safe, well tolerated and can be performed in an outpatient setting [5–12], the published literature on image-guided PLB is quite heterogeneous in terms of patient profile, underlying hepatic pathology, biopsy-related technical features and definitions of complications, making a direct comparison between studies difficult. In a cancer population, the possibility of tumour seeding has to be considered besides general complications such as post-biopsy bleeding. Only a few studies [13–15] have investigated the risk and frequency of tumour seeding after PLB using different biopsy techniques. The coaxial liver biopsy technique (C-PLB), which involves placing a larger outer sheath needle into the target tissue, through which subsequent biopsies are taken using a slightly smaller needle, has been proposed to decrease the risk of tumour seeding and complications in liver biopsies [14]. To date, no direct comparative study between a C-PLB and NC-PLB in an oncological setting has been performed. Therefore, the purpose of this paper was to assess the diagnostic yield, safety and seeding risk of a C-PLB versus a NC-PLB technique in a cancer patient cohort.

## Materials and methods

### Patients

Approval for this single-centre retrospective study was obtained from the local research committee and informed consent for the study was waived. A search through the clinical database was performed to identify all consecutive patients that had undergone an ultrasound or CT-guided liver biopsy between November 2011 and December 2017.

### Procedure

All patients provided written informed consent for the liver biopsy to be performed. Several pre-procedural safety measures were undertaken. Patient blood count should demonstrate a minimum platelet count of 60,000/mm^3^ and an International Normalized Ratio (INR) < 1.5. If patients were on anticoagulant drugs, these were stopped at least 24 h in advance prior to the blood tests, and any allergies were documented.

An ultrasound of the liver was first performed to identify any accessible and targetable lesions, which would be biopsied under ultrasound guidance. If no such lesion was identified, the procedure would be performed under CT guidance owing to the technique’s higher detection rate of liver lesions. Intravenous analgesic (fentanyl 25–100 μg) with or without sedation (midazolam 1–4 mg) were administered by a trained Interventional Radiology nurse to increase patient comfort; oxygen saturation, heart and respiratory rate were continuously monitored during the procedure.

Depending on the operator’s preference and the indication, PLB would be performed using a disposable fully automatic 18-gauge (G) or 16G core biopsy needle with or without a coaxial system (True-Core II, Argon Medical Devices). Under direct image guidance, a 17G or 15G coaxial needle was placed at the edge of the lesion and multiple cores were obtained with a 18G or 16G automatic core biopsy needle (True-Core II, Argon Medical Devices) respectively. Different areas inside the lesion were sampled by changing the angle and position of the coaxial needle. After the samples were collected, one to four preformed 18G or 16G gelatin foam pledgets (Hunter biopsy sealing device, Vascular solutions, Inc.) were deployed through the coaxial along the tract of the needle to facilitate haemostasis. After the completion of the procedure, the patient was transferred to radiology recovery for 4 h of bed rest and observation. Patients would leave the hospital the same day if no complications were reported.

### Data collection

Data analysis consisted of review of the patient records and re-review of the post-procedure imaging. In our institution, CT imaging is routinely performed at 8–12-week intervals to assess disease status. Demographics, primary tumour, indication for the biopsy, details of the PLB procedure and the occurrence and nature of complications (if any) following the Society of Interventional Radiology (SIR) Adverse Event classification [16] as well as the diagnostic yield and histopathological results were recorded. A good diagnostic yield was defined as when sufficient tissue materials were obtained to enable a definite histopathological diagnosis or molecular assay. All available follow-up imaging was investigated for signs of tumour seeding, defined as a visible small tumour deposit along the biopsy track. Cases in which seeding was suspected were re-reviewed by two board-certified radiologists (K.D.P and N.F.), and a consensus decision was made on the presence of seeding. As the course of the biopsy needle was no longer visible on follow-up imaging, this was estimated by correlating the presence of subcutaneous, subcapsular or anterior perihepatic soft tissue with the documented site of biopsy in the ultrasound biopsy report while considering associated technical considerations (e.g. supine or lateral decubitus position, intercostal approach).

### Statistical analysis

Statistical analysis comprised descriptive statistics to summarise the frequency of findings, especially with regard to diagnostic yield, complications and seeding. Complications were assessed on a per-biopsy level, while seeding was assessed on a per-patient level, only including patients with a histopathological confirmed malignancy and with follow-up imaging.

To compare both PLB techniques, non-parametric Mann-Whitney *U* test was performed to assess differences in achievable number of cores and Pearson’s chi-squared test to investigate differences in complication and seeding rate per technique and per needle diameter. Due to the high disease-related mortality number in our cohort, survival analysis was not performed as deemed not meaningful. The statistical package used was SPSS 22.0 for Windows and a *p* value < 0.05 was considered statistically significant.

## Results

### Patient demographics

During the study period, 741 patients underwent 932 PLB in total, the characteristics of which are summarised in Table 1. All patients were considered to have inoperable disease at the time of the biopsy. Eventually though, six patients ended up having a liver resection due to downstaging of the disease after chemotherapy (4 patients) or due to the biopsy result showing a second primary in the liver (2 patients). One patient had a liver transplant for multifocal hepatic haemangioendothelioma.

**Table 1:** Patient/biopsy characteristics

Biopsy number	932
Number of patients	741
Age	
Mean ± SD (years)	62 ± 13
Gender	
Male Female	49.0% (363/741) 51.0% (378/741)
Reason for biopsy	
Clinical trial Diagnosis/molecular	36.8% (343/932) 63.2% (589/932)
Biopsy technique	
Multiple passes Coaxial system	27.1% (253/932) 72.9% (679/932)
Diagnosis	
Negative Benign Malignant	7.4% (69/932) 3.0% (26/863) 97.0% (837/863)
Tumour type	
Colorectal Breast Lung Cholangiocarcinoma Pancreas Upper Gastrointestinal Prostate Melanoma Gynaecological Sarcoma* Urinary Head/neck Haematological Other	20.4% (151/741) 15.5% (115/741) 9.0% (67/741) 8.2% (61/741) 6.6% (49/741) 5.7% (42/741) 5.4% (40/741) 4.6% (34/741) 3.5% (26/741) 2.7% (20/741) 2.3% (17/741) 1.8% (13/741) 1.6% (12/741) 1.2% (9/741)

*SD* standard deviation

*Includes gastrointestinal stromal tumour (GIST, *n* = 9), and miscellaneous sarcoma types (*n* = 11)

Most patients (81.5%; 604/741) underwent only one PLB; however, 137 (18.5%; 137/741) had a PLB at several time points. Two PLBs were performed in 108 patients (14.6%; 108/741), three in 18 patients (2.4%; 18/741), four in three patients (0.4%; 3/741), and ≥ 5 in eight patients (1.1%; 8/741). Repeat biopsies were performed in accordance with clinical trials protocols or were clinically directed.

The indication for PLB was to obtain a (histopathological and/or molecular) tissue diagnosis in 63.2% (589/932) or as part of a clinical trial in 36.8% of cases (343/932). Only a minority of cases were performed under CT guidance (9.1%; 85/932), while the majority of biopsies were feasible under ultrasound guidance (90.9%; 847/932). A coaxial system was used in 72.9% (679/932) of PLB (C-PLB) and a non-coaxial technique (NC-PLB) in 27.1% (253/932). Both techniques predominantly used an 18G biopsy needle (69.1% (469/679) C-PLB, 85% (215/253) of NC-PLB). A significantly higher number of cores were obtained with C-PLB compared with NC-PLB (median = 4 (range 1–12) versus median = 2 (range 1–5); *p* < 0.001). Following biopsy, median patient follow-up time was 7 months (range 0–71 months), and post-PLB follow-up imaging was available for 630 of 656 patients (96%).

### Diagnostic yield

Only 69 of 932 PLBs (7.4%) were negative due to sampling error or inadequate due to insufficient tissue. There were no significant differences regarding diagnostic yield between biopsy techniques (C-PLB diagnostic in 92.6% (629/679), NC-PLB in 92.5% (234/253); *p* = 0.940) or needle diameters (18G diagnostic in 92.4% (632/684) and 16G in 93.1% (231/248); *p* = 0.70). A malignancy was demonstrated in 97.0% of PLBs (837/863) or 88.5% of patients (656/741). Most common tumour types encountered were colorectal (20.4%; 151/741), breast (15.5%; 115/741) and lung (9.0%; 67/741) cancers.

### Complications

A low overall complication rate of 9.3% (87/932) was noted; the most common complications per biopsy technique are summarised in Table 2. Complications were significantly less common when a C-PLB was used (8.2% (56/679) C-PLB versus 12.3% (31/253) NC-PLB; *p* = 0.024), regardless of needle diameter (18G 8.5% (58/684) complication rate, 16G 7.3% (18/248); *p* = 0.547). There were no procedure related deaths. The most common reported issues were mild grade I complications, such as pain (4.5%; 42/932) or transient hypotension (2.1%; 20/932). These were easily resolved by respectively increasing the pain medication, and the administration of fluids or atropine (0.3–1 mg IV). In a limited number of cases (2.5%; 23/932), however, patients needed admission in the hospital owing to more severe complications such as bleeding (1.2%; 11/932), sepsis (1.0%; 9/932), pulmonary embolism (0.2%; 2/932), pneumothorax (0.1%; 1/932) or deranged liver function tests (0.1%; 1/932). Eleven patients (1.2%; 11/932) presented with a post-procedural bleeding and had a median prolonged hospital stay of 1 day (range 1–18 days). Transarterial catheter embolisation was required in only one patient in whom a NC-PLB was performed, while 2 patients needed thorax drainage owing to haemothorax (NC-PLB and C-PLB). The rest of the patients presenting with complications were managed conservatively, including blood transfusion in five patients.

**Table 2:** Complications per biopsy technique

Complications	All	Non-coaxial	Coaxial
Overall	9.3% (87/932)	12.3% (31/253)	8.2% (56/679)
Grade 1 (mild)			
*Pain* *Hypotension*	4.5% (42/932) 2.1% (20/932)	7.5% (19/253) 2.0% (5/253)	3.4% (23/679) 2.2% (15/679)
Grades 2–4			
*Pneumothorax* *Pulmonary embolism* *Increased LFTs* *Sepsis/fever* *Bleeding*	0.1% (1/932) 0.2% (2/932) 0.2% (2/932) 1.0% (9/932) 1.2% (11/932)	0 0 0 1.6% (4/253) 1.2% (3/253)	0.1% (1/679) 0.3% (2/679) 0.3% (2/679) 0.7% (5/679) 1.2% (8/679)

*LFTs* liver function tests

### Seeding risk

**Fig. 1:**
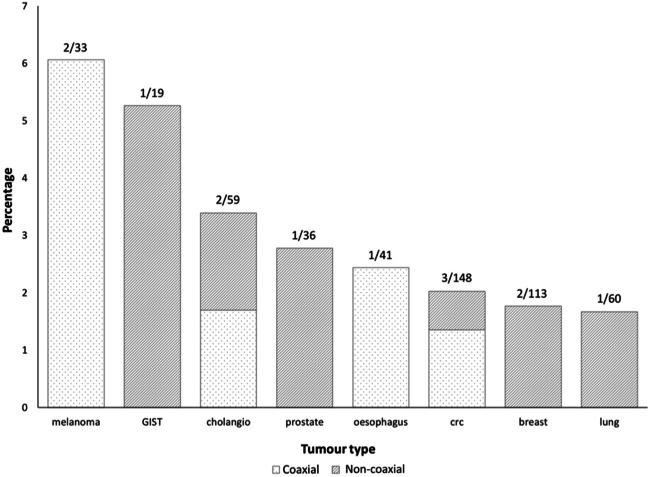
Seeding rate per tumour type relative to tumour prevalence

**Fig. 2:**
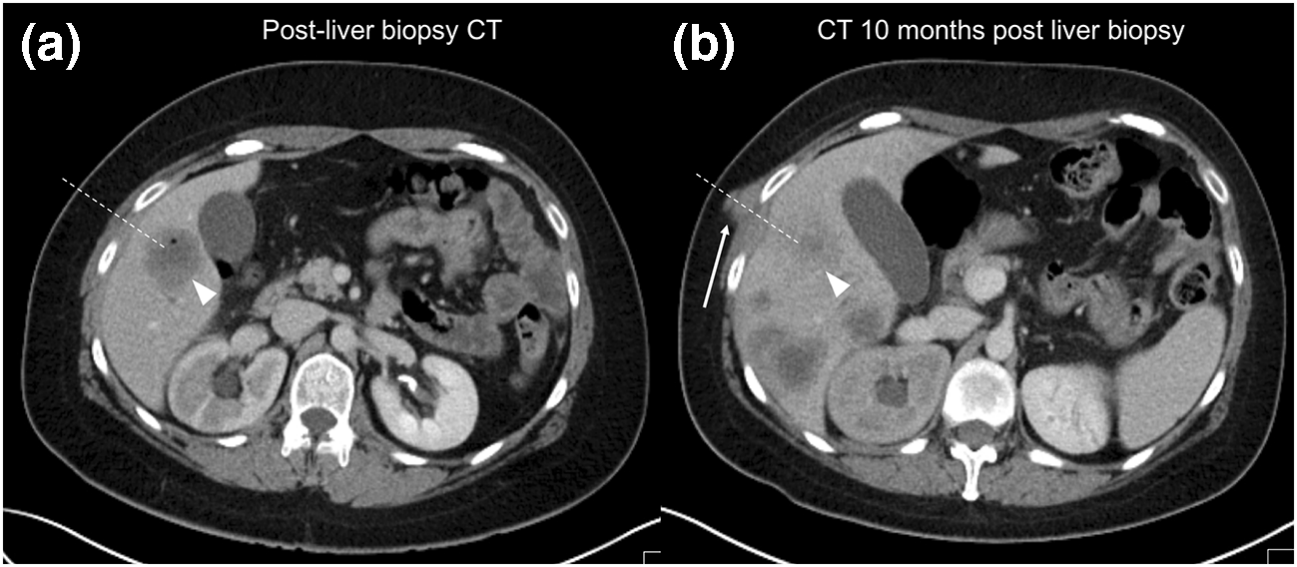
31-year-old man with metastatic colorectal cancer. **a** Staging CT performed immediately after an ultrasound-guided percutaneous liver biopsy of a lesion in the segment V of the liver (*white arrowhead*, *note gas bubble from biopsy*), which was performed by using a non-coaxial biopsy technique. **b** On the CT performed 2 months later, there are multiple new lesions in keeping with progressive disease. Furthermore, a new deposit (*white arrow*) is noted in the chest wall, along the needle track (*dotted white line*), indicating tumour seeding

Seeding was a rare event, occurring in only 13 of 741 patients (1.8%) with adequate imaging follow-up after a median time of 3 months (range 0–15 months) in different cancer types: 3/148 (2.0%) colorectal (CRC); 2/33 (6.1%) melanoma, 2/113 (1.8%) breast, 2/59 (3.4%) cholangiocarcinoma; 1/19 (5.3%) gastrointestinal stromal tumour (GIST), 1/41(2.4%) oesophageal, 1/36 (2.8%) prostate and 1/60 (1.7%) lung. Figure 1 shows that, relative to the tumour type prevalence in the current cohort, breast and lung cancers had the lowest seeding rate and melanoma the highest. Seeding occurred significantly (*p* = 0.021) more often in those patients in whom a NC-PLB was performed (3.0%; 6/197) compared with those who had had a C-PLB (1.3%; 7/544). The diameter of the biopsy needle did not affect the seeding risk (seeding risk 18G 1.7% (10/586) and 16G 1.9% (3/155); *p* = 0.847). Signs of seeding were only picked up at re-review of the images and not mentioned in any of the original imaging reports or the clinical notes as these were shown as very subtle lesions in the context of extensive disease progression elsewhere (Fig. 2).

## Discussion

Image-guided PLB plays a pivotal role in clinical cancer practice, enabling the collection of sufficient tissue for diagnostic purposes and advanced tissue analyses. In the realm of precision medicine, more and larger cores, often obtained before and at different time points during treatment, are required to perform the vast amount of genomic, transcriptomic and immunohistochemical analyses inherent to this field [4]. In this study, we showed that the use of C-PLB allowed for significantly more tissue sampling with fewer complications and a lower seeding rate than NC-PLB.

The diagnostic accuracy of both C-PLB and NC-PLB achieved the UK national audit standard of > 90% [11], with no significant difference between either technique.

Regarding complications, pain was the most frequently reported complaint as expected [12, 17]. Patients experienced less pain after C-PLB than NC-PLB and this is probably directly attributable to the lower number of passes through the liver capsule, reducing capsular pain. In line with previous large cohort studies [12], major complications were uncommon. For instance, a post-biopsy bleeding occurred only in 1.2% of cases, which is comparable with the results of Cui et al [5], who reported a bleeding risk of 1.43% in a cohort of 26,941 cancer patients across the USA. In this and most other studies, the impact of procedural factors such as biopsy technique, number of passes and needle size was not assessed. The single study comparing bleeding rates between a coaxial and non-coaxial biopsy technique [18], the latter with and without gel foam plugs, could not show a significant difference in complication rate. However, two cases of major bleeding necessitating embolisation and resulting in one patient’s death occurred after an unplugged biopsy. Similarly, the one patient requiring arterial embolization (0.1%) for bleeding in our study underwent a NC-PLB. In addition, a large prospective study [19] showed that the risk of bleeding rises with an increasing number of multiple passes. Hence, we believe that C-PLB with Gelfoam plugs does have its merit to decrease the risk of serious post-biopsy bleeding by minimising the number of capsular passes while still allowing for more tissue collection. Pulmonary embolism is an infrequent but major complication and occurred more often in our study than in previous reports. Most likely, this is attributable to the hypercoagulability inherent to cancer, putting patients at higher risk of thromboembolic events [20], in combination with the cessation of anticoagulation prior to the procedure.

Our study is the first and the largest to date to specifically and systematically evaluate the risk of tumour seeding post-PLB in cancer patients with a variety of histopathological aetiologies. Seeding was uncommon in the current study, occurring in only 13 out of 630 patients with imaging follow-up (2%) and only observed in the context of significant disease progression elsewhere in these patients. Even then, lesions along the biopsy track were very small and, in fact, only picked up on re-reviewing the images and none were diagnosed prospectively in the original radiology reports. This suggests that seeding may not have been the driving force for disease progression in patients having a biopsy of metastatic liver lesions. The limited number of studies investigating the seeding risk after PLB of metastases focused on breast and colon cancer [13, 15, 21, 22]. Chen et al [15] who evaluated all post-biopsy imaging in a cohort of 433 PBL of colon and breast metastasis found that none of the breast cancer patients presented with seeding, yet a 6% (17 of 278 patients) seeding rate was seen after PLB of colorectal metastases. In older retrospective studies comprising less than 51 patients, the risk of seeding post-PLB of colorectal metastasis was even higher ranging 10–16% [13, 21, 22]. These studies mainly sampled tissue by fine-needle aspiration and NC-PLB. In contrast to these results, we only found seeding in 3 out of 151 (2%) colorectal cancer patients after PLB. Instead, the seeding cases occurred in other tumour types, with a relatively higher ratio of occurrence in melanoma and GIST. We hypothesise the biopsy technique used may be crucial in decreasing tumour seeding along biopsy tracts. Using a coaxial system, there is only one penetration of the liver capsule with no direct contact with the metastasis when placing the coaxial needle at the edge of the target lesion. The fully automatic core biopsy needle samples the lesion and is retracted through the coaxial needle which protects the liver parenchyma and the soft tissues from tumour seeding. The risk of seeding in our study was significantly lower in those patients where a coaxial needle system was used. This is supported by the findings of Maturen et al [14], who did not have a single case of seeding after the use of a coaxial needle system for PLB of hepatocellular carcinoma (HCC), while a previous meta-analysis [23] found an overall seeding risk of 2.7% post-PLB in HCC.

Our study has several limitations. Firstly, this was a retrospective study and therefore there may have been a selection bias. However, all consecutive liver biopsies performed in our institutions in the given period were included in the analysis. Secondly, the choice of the biopsy technique reflected the radiologist preference and results may have been influenced by individual performances. Nevertheless, all biopsies were performed by experienced operators in both groups. Thirdly, the vast majority of the patients received systemic anticancer treatment post-liver biopsy which may have delayed or eliminated the occurrence of tumour seeding. Also, only a few patients had oligometastatic disease, and therefore, it’s not possible to estimate the seeding risk and its impact on survival in patients on a potential curative pathway. Nevertheless, our results do reflect standard oncology practice and the standard of care in our institution can be expected worldwide. Fourthly, no histological confirmation of suspected seeding lesions was obtained. As these were usually seen in a context of generalised progressive disease, sampling of these lesions would result in additional stress for patients with no added value to the clinical management, and was therefore deemed unethical. Finally, as the biopsy track is not identifiable on subsequent imaging, the course of the needle was estimated based on lesion location and available procedural imaging and reports. We tried to define the maximum possible seeding rate accepting that all lesions identified in the biopsy track were true positives, although some could actually represent disease progression. Therefore, the seeding rate in our study could be slightly overestimated, which only underscores the fact that seeding is very uncommon and should not be reason not to perform a PLB.

In conclusion, we found that C-PLB is of particular interest in the oncological setting and for clinical cancer trials. More and larger tissue cores can be obtained repeatedly in the same patient in safely manner with a lower risk of complications and seeding than NC-PLB.

## Abbreviations

CRC: Colorectal
CT: Computed tomography
GIST: Gastrointestinal stromal tumour
HCC: Hepatocellular carcinoma
INR: International Normalized Ratio
IV: Intravenous
PLB: Percutaneous liver biopsy
SIR: Society of Interventional Radiology

## References

[1] Aronson SJ, Rehm HL (2015). Building the foundation for genomics in precision medicine. Nature.

[2] Zardavas D, Piccart-Gebhart M (2015) Clinical trials of precision medicine through molecular profiling: focus on breast cancer. Am Soc Clin Oncol Educ Book 35:e183–e190 10.14694/EdBook_AM.2015.35.e183 25993171

[3] Khan K, Rata M, Cunningham D (2018). Functional imaging and circulating biomarkers of response to regorafenib in treatment-refractory metastatic colorectal cancer patients in a prospective phase II study. Gut.

[4] Hoang NS, Ge BH, Pan LY (2018). Determining the optimal number of core needle biopsy passes for molecular diagnostics. Cardiovasc Intervent Radiol.

[5] Cui Z, Wright JD, Accordino MK (2016). Safety, utilization, and cost of image-guided percutaneous liver biopsy among cancer patients. Cancer Invest.

[6] Janes CH, Lindor KD (1993). Outcome of patients hospitalized for complications after outpatient liver biopsy. Ann Intern Med.

[7] Froehlich F, Lamy O, Fried M, Gonvers JJ (1993). Practice and complications of liver biopsy. Dig Dis Sci.

[8] Perrault J, McGill DB, Ott BJ, Taylor WF (1978). Liver biopsy: complications in 1000 inpatients and outpatients. Gastroenterology.

[9] Van Thiel DH, Gavaler JS, Wright H, Tzakis A (1993). Liver biopsy. Its safety and complications as seen at a liver transplant center. Transplantation.

[10] Thampanitchawong P, Piratvisuth T (1999). Liver biopsy:complications and risk factors. World J Gastroenterol.

[11] Howlett DC, Drinkwater KJ, Lawrence D, Barter S, Nicholson T (2012) Findings of the UK national audit evaluating image-guided or image-assisted liver biopsy. Part I. Procedural aspects, diagnostic adequacy, and accuracy. Radiology 265:819–831 10.1148/radiol.12111562 23175545

[12] Howlett DC, Drinkwater KJ, Lawrence D (2013). Findings of the UK national audit evaluating image-guided or image-assisted liver biopsy. Part II Minor and major complications and procedure-related mortality. Radiology.

[13] Jones OM, Rees M, John TG, Bygrave S, Plant G (2005) Biopsy of resectable colorectal liver metastases causes tumour dissemination and adversely affects survival after liver resection. Br J Surg 92:1165–1168 10.1002/bjs.4888 15997444

[14] Maturen KE, Nghiem HV, Marrero JA (2006). Lack of tumor seeding of hepatocellular carcinoma after percutaneous needle biopsy using coaxial cutting needle technique. AJR Am J Roentgenol.

[15] Chen I, Lorentzen T, Linnemann D (2016). Seeding after ultrasound-guided percutaneous biopsy of liver metastases in patients with colorectal or breast cancer. Acta Oncol.

[16] Khalilzadeh O, Baerlocher MO, Shyn PB (2017). Proposal of a new adverse event classification by the Society of Interventional Radiology standards of practice committee. J Vasc Interv Radiol.

[17] Maheux A, Purcell Y, Harguem S, Vilgrain V, Ronot M (2019) Targeted and non-targeted liver biopsies carry the same risk of complication. Eur Radiol. 10.1007/s00330-019-06227-3 10.1007/s00330-019-06227-3 31076864

[18] Hatfield MK, Beres RA, Sane SS, Zaleski GX (2008). Percutaneous imaging-guided solid organ core needle biopsy: coaxial versus noncoaxial method. AJR Am J Roentgenol.

[19] Atwell TD, Smith RL, Hesley GK (2010). Incidence of bleeding after 15,181 percutaneous biopsies and the role of aspirin. AJR Am J Roentgenol.

[20] Falanga A, Marchetti M, Vignoli A (2013). Coagulation and cancer: biological and clinical aspects. J Thromb Haemost.

[21] Ohlsson B, Nilsson J, Stenram U, Åkerman M, Tranberg K‐G (2002) Percutaneous fine-needle aspiration cytology in the diagnosis and management of liver tumours. Br J Surg 89:757–762 10.1046/j.1365-2168.2002.02111.x 12027987

[22] Rodgers MS, Collinson R, Desai S, Stubbs RS, McCall JL (2003) Risk of dissemination with biopsy of colorectal liver metastases. Dis Colon Rectum 46:454–458 discussion 458-9 10.1007/s10350-004-6581-6 12682536

[23] Silva MA, Hegab B, Hyde C, Guo B, Buckels JAC, Mirza DF (2008) Needle track seeding following biopsy of liver lesions in the diagnosis of hepatocellular cancer: a systematic review and meta-analysis. Gut 57:1592–1596 10.1136/gut.2008.149062 18669577

